# Evaluation of computational programs to predict HLA genotypes from genomic sequencing data

**DOI:** 10.1093/bib/bbw097

**Published:** 2016-10-31

**Authors:** Denis C Bauer, Armella Zadoorian, Laurence O W Wilson, Melbourne Genomics Health Alliance, Natalie P Thorne

**Affiliations:** 1 CSIRO, Sydney, Australia; 2 School of Biotechnology and Biomolecular Sciences, University of New South Wales, Sydney, Australia; 3 Murdoch Childrens Research Institute, Royal Children’s Hospital, Parkville, Australia; 4 Department of Medical Biology, The University of Melbourne, Parkville, Australia; 5 Melbourne Genomics Health Alliance, Parkville, Australia; 6 Walter and Eliza Hall Institute, Parkville, Australia

**Keywords:** HLA genotyping, in silico pathology test, NGS, clinical genomics

## Abstract

**Motivation:**

Despite being essential for numerous clinical and research applications, high-resolution human leukocyte antigen (HLA) typing remains challenging and laboratory tests are also time-consuming and labour intensive. With next-generation sequencing data becoming widely accessible, on-demand *in silico* HLA typing offers an economical and efficient alternative.

**Results:**

In this study we evaluate the HLA typing accuracy and efficiency of five computational HLA typing methods by comparing their predictions against a curated set of > 1000 published polymerase chain reaction-derived HLA genotypes on three different data sets (whole genome sequencing, whole exome sequencing and transcriptomic sequencing data). The highest accuracy at clinically relevant resolution (four digits) we observe is 81% on RNAseq data by **PHLAT** and 99% accuracy by **OptiType** when limited to Class I genes only. We also observed variability between the tools for resource consumption, with runtime ranging from an average of 5 h (**HLAminer**) to 7 min (**seq2HLA**) and memory from 12.8 GB (**HLA-VBSeq**) to 0.46 GB (**HLAminer**) per sample.

While a minimal coverage is required, other factors also determine prediction accuracy and the results between tools do not correlate well. Therefore, by combining tools, there is the potential to develop a highly accurate ensemble method that is able to deliver fast, economical HLA typing from existing sequencing data.

## Introduction

**Figure 1:**
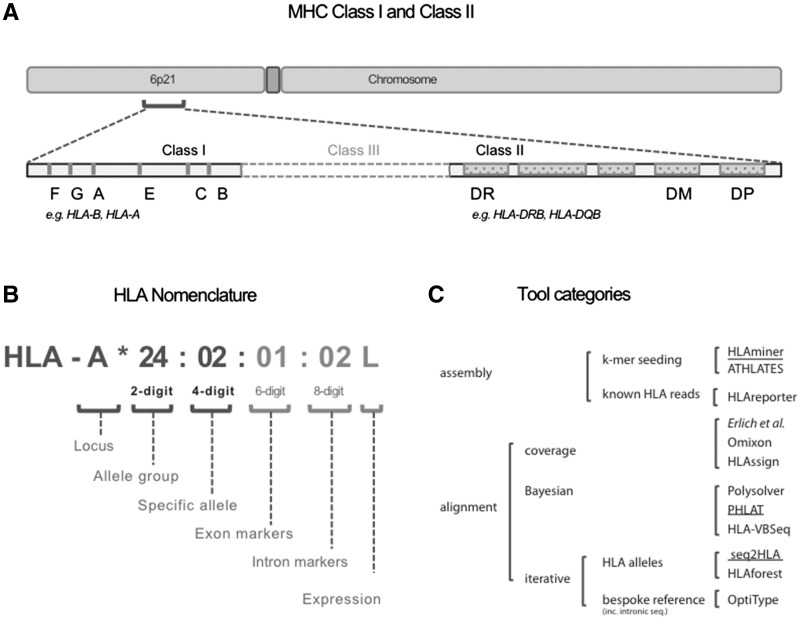
Genomic location and nomenclature. (**A**) Genomic location of the HLA Class I and II genes. (**B**) Nomenclature of HLA Alleles. (**C**) Tools grouped by category as discussed in the text. Underlined names denote tools designed for RNA and DNA data.

Widely used in both clinical and research contexts, accurate identification of a person’s human leukocyte antigen (HLA) allele is necessary for many applications. However, owing to the inherent highly polymorphic nature of the HLA system, and the lack of a known complete sequence of this chromosome 6p21.3 region (see Figure 1A), HLA typing remains challenging [1–3]. As such, better HLA typing approaches overcoming these issues and offering rapid, inexpensive and high-throughput genotyping are needed. Correspondingly, with the advent of next-generation sequencing (NGS), computational tools capable of genotyping HLA using either whole genome (WGS), whole exome (WES) or transcriptomic sequencing (RNAseq) data as input demonstrate immense potential for satisfying these needs and becoming the new more practical gold standard approach for HLA typing.

Being the human major histocompatibility complex, the HLA system encodes cell surface residing glycoproteins involved in self and foreign protein recognition and immunity [4]. In particular, the HLA Class I (HLA-A, -B, -C) and Class II (HLA-DP, -DQ, -DR) molecules, respectively, present endogenous antigens to CD8+ cytotoxic T cells for cell destruction, and exogenous antigens to CD4+ helper T cells for antibody production [5, 6]. Each of these genomic loci can have a number of different alleles comprising multiple specific variants observed in the genomic sequencing (see Figure 1B).

The nomenclature for describing HLA alleles uses a hierarchical numbering system differentiating allele groups (two-digit accuracy) from specific alleles (four-digit accuracy). Increasing the resolution, the exonic variants (six digits) that are not associated with allele groups are distinguished from the intronic variants (eight digits). The nomenclature also allows a tag to be added to flag alleles with observed low (L) or questionable (Q) expression. However, as noted by Marsh *et al.* [7], it has become increasingly difficult to consistently link sequence-derived allele names to serology-derived results. These difficulties are in part technological, e.g. synonymous mutations without serology effect, and in part owing to the observation that some newly defined antigens do not comfortably fit within any known serological grouping. Specifically, the HLA-DRB1*03, *11, *13, *14 and *08 family of alleles exhibit a continuum of allelic diversity rather than five discrete sub-families. A person’s HLA genotype is formed by using the nomenclature to specify the alleles from both chromosomes at a given HLA locus.

HLA typing has been widely used for reducing the risk of organ rejection and graft-versus-host disease when matching donors and recipients of solid organ and allogenic haematopoietic stem cell transplants [1, 8]. Specific HLA variants are associated with both autoimmune (e.g. type 1 diabetes, rheumatoid arthritis) and infectious (e.g. HIV, Hepatitis C) diseases [9], and adverse drug reactions such as Stevens–Johnson syndrome and toxic epidermal necrolysis [10, 11]. HLA typing is used for aiding the diagnosis as well as drug prescription choices of these conditions.

The current laboratory-based approaches used for HLA typing evolved from serology-based techniques that rely on the binding of unknown HLA antigens to known antibodies. They enable low, two-digit antigen resolution typing [12, 13]. With the development of the polymerase chain reaction (PCR), the HLA genes—namely, exons 2 and 3 of HLA Class 1, and exon 2 of HLA Class 2 genes [9, 14]—could be amplified and quantified via sequence-specific oligonucleotide probes (SSOPs) or primers [15, 16] or PCR amplified followed by Sanger sequencing, with the resulting sequence compared against the HLA reference (SBT). SBT is currently considered the gold standard. However, while these molecular genotyping approaches enable better resolution typing than their serological counterparts, they are still expensive, labour intensive and time-consuming methods that do not meet the high-throughput requirements of clinical and research contexts [14, 17, 18].

Flow cytometry and loop-mediated isothermal amplication (LAMP) are recent HLA typing laboratory methods that have been developed but they lack resolution and accuracy, respectively. Specifically, LAMP, being a primer-based approach, cannot accurately detect rare or novel alleles [19]. SNP arrays, being primer-based approaches, suffer the same limitation, and as such, computational methods using array data to predict HLA such as SNP2HLA [20] also have poor accuracy.

The recently developed (2012–2015) computational programs that type HLA from NGS data overcome many of these challenges. For patients who have already had a genomic test, HLA typing by re-analysis of their NGS data would avoid time and costs associated with performing a separate laboratory test. Furthermore, sequencing-based approaches can type HLA alleles on each homologous chromosome (chromosome-specific alleles) such that heterozygous alleles can be detected, and polymorphisms outside the traditionally amplified PCR regions can be detected to allow higher resolution typing [9, 21]. However, these approaches are also limited by read length and coverage insufficiencies, and the highly polymorphic nature of the HLA system [1, 8, 18]. To overcome these challenges, NGS-based *in silico* HLA typing methods use a variety of different techniques that fall into two broad categories (see Figure 1C), alignment- or assembly-based methods. Alignment-based methods align sequencing reads against reference HLA sequences (genomic, exomic or transcriptomic) and predict true alleles based on probabilistic models. *De novo* assembly-based approaches assemble reads into contigs and align these to the reference sequences of known HLA alleles.

Erlich *et al.* [22] first described the use of NGS data (Roche 454) to perform HLA typing. Their method saw reads aligned (using the SSAHA2 aligner) against the International ImMunoGene Tics (IMGT)-HLA database [23] followed by a quality filter step to remove reads with high alignment error rate. Genotypes were determined based on the resulting coverage of the HLA alleles in the database. Wang *et al.* [24] developed a similar approach using BLASTN as the aligner with a more stringent filter criterion (e.g. valid distance between read pairs) but also extended the approach by *de novo* assembling contigs (EZ_assembler) and comparing the resulting sequences with the known reference to detect novel alleles.

Both studies did not release the source code but the code is available for the similar alignment-filter-coverage approaches from **Omixonsoftware** [25], which allows fewer mismatches and insertions, and **HLAssign** [14], which introduces different filtering criteria. The most recently published alignment-based methods, **Polysolver** [26] and **HLA-VBSeq** [27], both adopt a Bayesian classification approach for determining the HLA genotype.

Developing the *de novo* assembly idea further, **HLAminer** [16] uses the read-assembly tool TASR to generate a *k*-mer table of the IMGT/HLA database, and uses these to identify seed reads for the subsequent assembly. The method is reported to work on DNA and RNA data. The resulting contigs are blasted against the IMGT/HLA reference and genotypes are chosen based on their score and probability for being observed given the contigs. They also released an alignment-only option, however found it to be inferior in their analysis.

A similar *k*-mer pre-filter approach was adopted by **ATHLATES** [18]; however, they first extract the exonic sequences from the IMGT/HLA database and choose the most probable HLA allele as the one with the minimal Hamming distance between the contig and each individual HLA allele. The most recent approach using assembly is **HLAreporter** [28], which first maps the reads to the IMGT/HLA reference sequence and then assembles the mapped reads into contigs. They then adopt the same scoring and HLA allele-calling as **HLAminer**.

The first HLA typing method specifically developed for RNA was **seq2HLA** [17]. The method maps reads to the MGT/HLA sequences and then in a greedy approach determines the allele with the highest number of mapped reads for each locus individually. After discarding the selected alleles and already assigned reads, second alleles are selected accordingly. The most recent RNA-optimized predictor, **HLAforest** [29], uses a tree-based top-down greedy algorithm to use the implicit hierarchy of HLA nomenclature. The algorithm generates for each read a tree denoting all the HLA alleles this read could be aligned to. Trees are collectively weighted by taking all reads into account and pruned to iteratively remove and re-weight leaf nodes until only the most likely leaf node remains.

Like **HLAminer**, **PHLAT** [1] was developed to use DNA or RNA data and builds on Erlich *et al.*’s alignment-filter-coverage approach; however, it then applies a likelihood model, which combines the probability of unevenly distributed HLA alleles in the human population with the likelihood of the observed coverage of the different alleles at each locus. **OptiType** [15] also predicts from DNA or RNA data; however, it leverages information from the intronic regions to make its calls. As 94.6% of HLA sequences contained in the IMGT database lack parts of their exonic or intronic sequences, they had to reconstruct the reference by imputing from the other partially sequenced alleles with small phylogenetic distance. The HLA allele is then determined by solving the optimization problem of finding the best combination of up to six major and six minor HLA alleles, which maximizes the number of reads mapped to this selection, under the biological constraints that at least one and at most two alleles are selected per locus. The method currently has only HLA Class I reference information available.

In this study, we compare these 12 HLA typing computational programs with the aim of evaluating their prediction performance, and potentially identifying an optimal HLA typing approach. To achieve this, we first compile a novel literature curated test set of samples with publicly available PCR-verified HLA genotypes (gold standard). For these samples we then source WGS, WES and RNAseq and compare the predictions made by the NGS tools against the HLA genotype in the gold standard. We evaluate the tools’ accuracy and efficiency on all data sets noting that some tools were specifically designed for DNA or RNA (only **OptiType**, **PHLAT** and **HLAminer** are designed to handle both) or Class I genes (**OptiType**).

## Methods

### Gold standard

To enable comparison of the prediction performance of recently developed NGS-based HLA typing tools, we created a literature curated data set consisting of >1300 samples from five published studies whose HLA genotypes have been determined using PCR-based methods (see Table 1). We collated the genotypes from all sources and annotated any discrepancies in the HLA alleles that were reported for each locus. We marked these disagreements as ‘conflicts’ and created a duplicate row in our data set for each different allele that was reported for each sample. For the evaluation we treated a prediction as correct if it corresponded to any of the reported alleles for the locus in the sample (see Results section).

**Table 1:** PCR-based method used in the gold standard data set

Data set	PCR method	Sample numbers
De Bakker *et al.* [32]	PCR-SSOP amplification followed by visualization hybridization patterns via autoradiography	229
Erlich *et al.* [22]	SO hybridization and exon sequencing using the Roche 454 GS FLX Titanium platform	12
Warren *et al.* [16]	PCR amplicons cloned and sequenced using an ABI 3730XL instrument (Class I only)	16
Liu *et al.* [18]	PCR amplification followed by Sanger sequencing of the exons (SBT)	13
Bai *et al.* [1]	PCR amplification followed by Sanger sequencing of the exons (SBT) (HLA-A and -B loci only)	5
Gourraud *et al.* [8]	PCR amplification followed by Sanger sequencing of the exons (SBT)	1233

**Figure 2:**
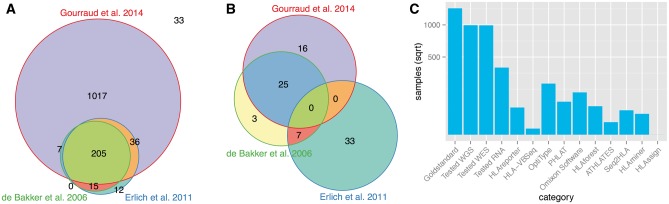
Gold standard. (**A**) Samples in common between different studies. The image does not show the 33 samples from Bai *et al.* (5), Liu *et al.* (13) and Warren *et al.* (16), as only three samples from Liu *et al.* intersect with the other studies. (**B**) Agreement in HLA typing of 42 samples where there were discordant results between at least two studies. (**C**) Total number of samples with HLA typing information and tested in this study, as well as the number of these samples used by the different prediction tools for development. Y-axis on square root scale.

Furthermore, in our gold standard data set, we also recorded whether these primary literature sources typed all or only some of the loci. Unless stated otherwise in Table 1, results for loci that are missing for some samples are likely owing to failed primer hybridization and we did not include those sample loci in the comparison. Moreover, we also made note of which samples were used to train and develop each tool (see Figure 2C).

### Fastq file preparation

From the 2706 samples in the 1000 Genomes phase 3 data set, we downloaded the WGS and WES alignment files from the 1147 samples with HLA typing in our gold standard table (European Nucleotide Archive ERP006600).

For 154 and 155 samples, no WGS and WES alignment data, respectively, was available. Our final data sets included 993 and 992 WGS and WES, respectively. We also downloaded from the 465 lymphoblastoid cell lines RNAseq data [30] (ERP001942) the 373 alignment files for which HLA typing information was available. For all three data sets, we extracted the aligned reads from the bam file using samtools/1.3 view for the region 6:29677984-33485635, combined them with the unaligned reads, and used bedtools/2.25.0 bamtofastq to convert them to fastq files.

#### Evaluation metric

Success rate is calculated over all alleles and all samples as

$$\text{Success}=\frac{\#\text{RightAlleles}}{\#\text{RightAlleles}+\#\text{WrongAlleles}}$$

where ‘Right Allele’ means the HLA allele called by the software tool matches any of the PCR-determined alleles provided by one or more literature sources for a specific locus. Typing ambiguity is a well-established phenomenon [31]. Three of the investigated programs hence suggest more than one allele for loci where typing confidence is low. We therefore calculate the ‘Approximate Success’ by taking into account the top 5 predictions for **HLA-VBSeq**, and the top 3 predictions (per chromosome) for **HLAminer** and **seq2HLA** and evaluate a call as approximately accurate if the correct solution is among this list.

The accuracy is also called over all samples but takes the number of uncalled alleles into consideration with

$$\text{Accuracy}=\frac{\#\text{RightAlleles}}{\#\text{RightAlleles}+\text{WrongAlleles}+\text{NAAlleles}}$$

Similar to ‘Approximate Success’, ‘Approximate Accuracy’ again is calculated over the top 5 and 3 predictions for **HLA-VBSeq** and **HLAminer**, respectively.

### Program execution notes

As listed in Table 2, not all published tools could be included in the comparison. This is because, despite seeking advice from the developers, we were unable to run several tools (**ATHLATES**, **HLAforest**, **HLAreporter**, **HLAssign**). We were able to directly execute **HLA-VBSeq**, **OptiType**, **PHLAT** and **seq2HLA** and updated **HLAminer**’s source code to support multi-sample experiments by enabling the output to be redirected into a sample-specific location.

**Table 2:** Overview of the computational HLA typing methods published to date

Tool name	Class	Resolution	Chrom-specific	Input	Method	Approach	Maintained	Tested	Data set	Self-reported two-digit accuracy (%)
Polysolver [26]	I+II	4 digits	Y	DNA	Alignment	Two-step Bayesian classification approach involving alignment by reads to IMGT reference; keeping best scoring alignment	Y	N^a^	253 HapMap WES	97
HLAreporter [28]	I+II	4 digits	Y	DNA	Alignment+ Assembly	Identifies HLA allele matches based on scoring system for assembled contigs.	Y	N^b^	82 WES from 1KG and HapMap	100
HLAssign [14]	I+II	6 digits	Y	DNA	Alignment	Coverage-based genotype of exclusively mapped read intended for use on in-solution targeted capturing	N	N^c^	357 cell lines	99
HLA-VBSeq [27]	I+II	8 digits	N	DNA	Alignment	Variational Bayesian and posterior distribution to optimize read alignments and scoring system for typing.	Y	Y	simulated data, CEU trio	100
OptiType [15]	I	4 digits	N	DNA/RNA	Alignment	Constructed hit matrix and used integer linear programming for the optimization.	Y	Y	253 1KG exome	97
PHLAT [1]	I+II	6 digits	Y	DNA/RNA	Alignment	Gaussian distribution for testing statistical significance of selected candidate alleles.	Y	Y	50 HapMap RNA, 10 1KG exome, 15 Hapmap exome	99
Omixonsoftware [25]	I+II	6 digits	Y	DNA	Alignment	Uses own formula for allowing mismatches, then successively discards alleles until it reports allele pairs containing high number of mapped reads and adequate exon coverage.	Y	N^d^	447 1KG genome, 217 1KG exome	90
HLAforest [29]	I+II	4 digits	Y	RNA	Alignment	Builds weighted alignment tree (generates own alignment probability).	N	N^e^	simulated data, own, 50 HapMap RNAseq, 16 CRC RNAseq	99
ATHLATES [18]	I+II	4 digits	Y	DNA	Assembly	Identifies candidate alleles based on their Hamming distance.	N	N^b^	16 1KG WES, 13 own	99
seq2HLA [17]	I+II	4 digits	Y	RNA	Alignment	Calculated variability at positions across exons 2 and 3 using Shannon’s entropy, and information content using binary logarithm formulation.	Y	Y	50 HapMap RNAseq and 37 own RNAseq	96
HLAminer [16]	I+II	4 digits	Y	DNA/RNA	Alignment or Assembly	Putative HLA alleles are characterized based on scoring system of assembled contigs.	Y	Y	simulated data; 16 own RNAseq; 20 HapMap	NA^f^
[24]	I+II	4 digits	NA	DNA	Alignment + Assembly	HLA typing based on coverage information of aligned reads supplemented by contig matching for unseen mutations	N	N^g^	40 cell lines, 59 WGS	99

Chrom-specific refers to the ability of the tool to predict the allele on each chromosome separately rather than returning the two most likely genotypes overall. 1000 Genomes abbreviated

^a^ Requires the commercial aligner Novoalign.

^b^ Code not executable (conversation with developer).

^c^ Code not executable (no reply from developer).

^d^ Limit on samples for free version.

^e^ Communication with developer: discontinued.

^f^ Reports sensitivity and specificity.

^g^ No code available.

## Results

### The concordance of HLA typing by the current gold standard methods is low

Our gold standard data set comprises PCR-based HLA typing data from several different studies that used samples from the 1000 Genomes study. Each study used different variations of PCR-based HLA typing methods, so that we could assess the agreement of typing methods between studies that used the same samples.

In Figure 2A, we summarize the samples in common between the different studies. Gourraud *et al.* [8] and Erlich *et al.* [22] have 1017 and 12 distinct samples, respectively. A further 33 samples in total were HLA typed in Bai *et al.* [1], Liu *et al.* [18] and Warren *et al.* [16], but not in any other studies. Of the 263 samples (205 + 15 + 7 + 36) that were HLA typed in more than one study, only 84% of these samples (*n* = 221) had HLA type results that agreed between the studies demonstrating the discrepancies that arise between different PCR-based methods. For the 42 samples where HLA type results disagreed between at least two studies, we investigated the concordance of HLA typing results between the three studies with the most samples in common (Figure 2B).

Most concordance can be seen between Gourraud *et al.* [8] and de Bakker *et al.* [32] likely owing to the underlying methodology being similar, i.e. de Bakker *et al.* [32] used PCR-SSOP amplification followed by visualizing hybridization patterns via autoradiography, and the more recent method by Gourraud *et al.* [8] used PCR amplification followed by Sanger sequencing of the exons (SBT). Erlich *et al.* [22], on the other hand, omitted the PCR amplification step and combined results from SSO hybridization with exon sequencing from the Roche 454 GS FLX Titanium platform.

This is somewhat surprising as Lane *et al.* [33] compare the HLA status derived from SSOP against clonally amplified DNA, using Roche 454 technology for 993 samples from newborns with maternally reported African American ancestry and found a concordance of 92.3%. However, it is consistent with Gourley *et al.* [34], who reported typing discrepancies between 3.9% and 6.7% for HLA-A and B, respectively, based on SSOP typing of 1983 samples. The discrepancy we observed in our gold standard data set may hence reflect inaccuracy of the laboratory method as well as inter-laboratory variability. Because there is an underlying ambiguity, we include all observed genotypes in the gold standard table and assess the computational tool against the union.

### PHLAT has the highest accuracy

We compare the runnable programs (see Methods) on the fastq files extracted from the HLA region of the 1000 Genomes alignment files and compare the tools’ predictions against the gold standard table as described in the ‘Methods’ section. The accuracy for the four-digit resolution prediction is listed in Table 3 (WGS), with more detailed tables (two digits, Class I) listed in the Supplementary Material (Supplementary Table S1–S3).

**Table 3:** Accuracy table NGS data for Class I + II

Data set (Samples)	Tool	Accuracy (Success)	**Approximate accuracy (Success)**	**Samples failed**
WGS	optitype^+^	35% (71%)		6
(993)	hlavbseq	**52%** (52%)	**66%** (66%)	0
	hlaminer assembly	17% (36%)	23% (49%)	19
	hlaminer alignment	15% (26%)	20% (35%)	0
	phlat	38% (46%)		0
	seq2hla*	7% (12%)	9% (32%)	0
WES	optitype^+^	49% (98%)		1
(992)	hlavbseq	68% (68%)	**77%** (77%)	0
	hlaminer assembly	43% (49%)	53% (61%)	0
	hlaminer alignment	26% (27%)	42% (43%)	0
	phlat	**73%** (73%)		0
	seq2hla*	60% (61%)	71% (71%)	0
RNA	optitype^+^	50% (99%)		0
(373)	hlavbseq*	67% (67%)	80% (80%)	0
	hlaminer assembly	52% (61%)	61% (71%)	0
	hlaminer alignment	20% (20%)	30% (30%)	0
	phlat	**81%** (81%)		0
	seq2hla	79% (79%)	**81% (81%)**	0

HLA typing results for four-digit resolution on 1000 Genomes Project samples. Bold highlights the best performance in the category.

‘*’ labels tools that were not designed to handle DNA or RNA data, respectively.

+ **OptiType** predicts Class I only, hence can only achieve an accuracy of 50%.

Please see Supplementary Tables S1–S3 for Class I comparison.

**Figure 3:**
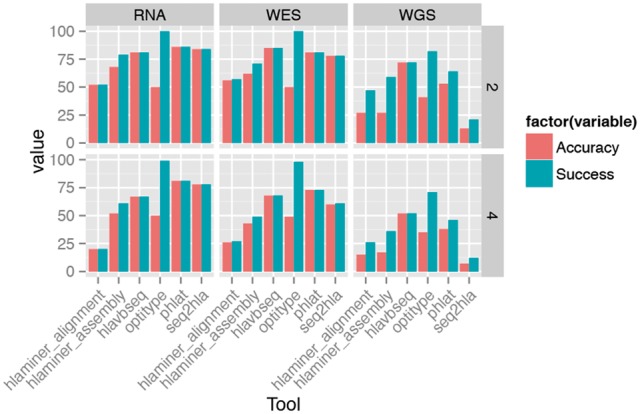
Accuracy and success rate for each tool for the three different data sets and two different resolutions (two and four digits).

As shown in Figure 3, the overall highest accuracy (81%) is achieved by **PHLAT** on the RNAseq data set. For WES, **HLA-VBSeq** performs the best (77%) when the correct solution in the top 5 predictions is accepted. When evaluating only the top solution, **PHLAT** performs the best with 73%. For WGS, the best performance is achieved by **HLA-VBSeq** both for the approximate (66%) and exact (52%) results. In our test, **HLAminer** consistently performs poorly, particularly using the alignment-based workflow. **OptiType** only predicts HLA Class I genotypes; however, it is the only tool in the test that predicts 99% (four digits) of Class I genotypes correctly. The next best performance on this subset was by **PHLAT** with 96% (four digits). We see similar results when the analysis is limited to the 37 samples that had a single genotype for every HLA locus (see Supplementary Figures S2 and S3).

### Coverage is not influencing performance

**Figure 4:**
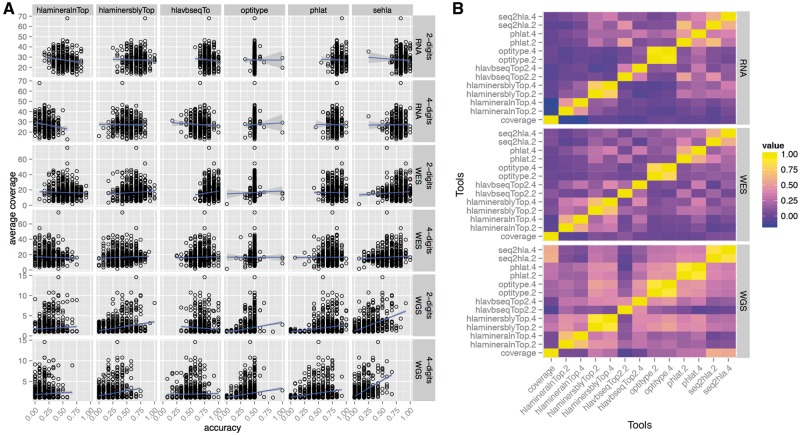
Association between coverage and accuracy. (**A**) Class I + II accuracy versus the average coverage over the HLA region (6:29677984-33485635) as mapped by Razers3 [35]. Note while **OptiType** only predicts Class I loci, the plot shows some samples reaching >50% owing to these samples lacking a PCR-determined Class II genotype. (**B**) Correlation of the prediction accuracy for each sample between the different tools as well as the read coverage in this sample.

Major *et al.* noted that phase 1 of the 1000 Genomes data was unsuitable for HLA typing, as the coverage was low. As shown in Supplementary Figure S1, phase 3 also has low coverage with only 2-fold coverage (Standard Error (ste) = 0.05), compared with WES with 17-fold coverage (ste = 0.22) and RNA 27-fold coverage (ste = 0.36). We therefore investigated the claim by Major *et al.* that coverage has a direct influence on accuracy. As shown in Figure 4A, there is only a weak Pearson correlation between accuracy and coverage, except for **seq2HLA** on WGS data (Correlation Coefficient *r* = 0.76). More generally, Figure 4B shows that the accuracy of the tools on the same sample are only weakly correlated (RNA mean = 0.17, ste = 0.02; WES mean = 0.23, ste = 0.02; WGS mean = 0.37, ste = 0.02), indicating that there are other factors influencing accuracy, which affect the tools differently.

### seq2HLA is the most performant

**Figure 5:**
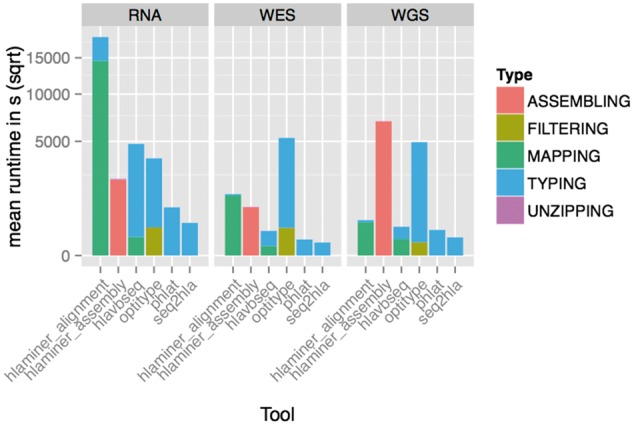
Runtime of the different tools showing the breakdown of different tasks. Y-axis on square root scale.

In this section, we discuss the resource consumption as recorded on a LINUX cluster of 64 Intel Haswell 10-core processors with 25 MB cache and 8 TB of global shared memory. Figure 5 visualizes the average runtime per tool and data set broken down by task.**seq2HLA** is the fastest program, with an average runtime of 65 s per sample (ste = 1.7 s) on WES, compared with 1 h 28 min per sample (ste = 27 s) by **OptiType** on the same data. **HLAminer** alignment does not use any parallelization strategies for alignment (bwa) nor typing and hence uses 5 h per sample (ste = 403.6 s) on RNAseq data compared with 7 min by **seq2HLA** (ste = 18.7 s). With the highest accuracy on RNAseq data, **PHLAT** requires on average 15 min (ste = 19.9 s) per sample.

**Figure 6:**
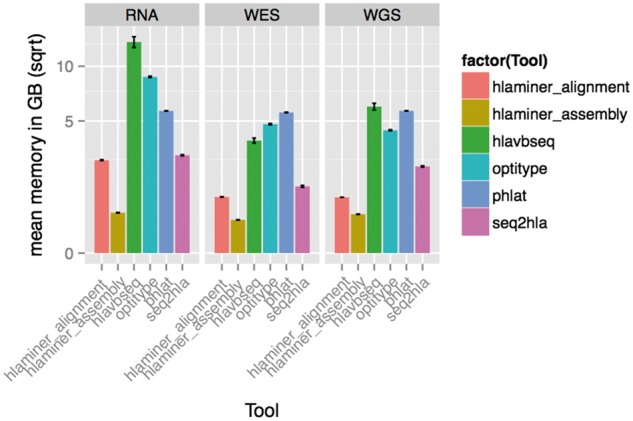
Memory consumption of the different tools. Y-axis on square root scale.

Figure 6 visualizes the average memory consumption per tool. **HLAminer** assembly has the lowest memory consumption with 0.3 GB (ste = 0.003) on WES, compared with 5.7 GB (ste = 0.03) by **PHLAT**. **HLA-VBSeq** has the largest consumption of memory on RNA data with 12.8 GB (ste = 0.65) compared with **HLAminer** assembly with 0.46 GB (ste = 0.007). **PHLAT** required on average 5.8 GB (ste = 0.0002) per sample.

Overall, **seq2HLA** is the most performant method with consistently the fastest runtime while simultaneously being among the three tools consuming the least amount of memory.

## Discussion and conclusion

In this article, we have evaluated publicly available computational tools for HLA typing from sequencing data for their accuracy and resource consumption on WGS, WES and RNAseq data. The majority of the published tools (7 of 12) could not be included in the comparison because the software was not executable in our hands, they depended on commercial components, imposed sample restrictions or were not made available. Of the remaining five tools, none delivered the high prediction accuracy claimed in the respective papers.

**OptiType** performed closest to the reported accuracy with 99% for four-digit resolution for RNAseq Class I loci (four-digit accuracy on WGS was 71% and WES 98%), see Supplementary Tables S1–S3. Although **OptiType** may have benefitted by seeing more of the test data than other tools (21%; see Figure 2C), the high accuracy of this method is more likely owing to their purposely constructed reference genome that uses a broader region of exonic and intronic sequence than the smaller PCR-based reference regions in the IMGT/HLA database.

Unfortunately, as **OptiType** can only type HLA Class I genotypes, its clinical application may be limited. The authors claim that ‘it can be easily adapted to predict genotypes for loci other than HLA-I such as HLA-II’; however, as there are fewer known alleles for Class II compared with Class I (3743 versus 10,730), there may be insufficient diversity captured yet to construct an equally high performing reference sequence for typing Class II genotypes with **OptiType**.

Class I + II predictions were most accurately made by **PHLAT** with 81% accuracy on four digits. **PHLAT** is also the second fastest tool with on average 4.8 min per sample (ste = 7.6 s) over all data sets but requires the second most memory with on average 5.7 GB per sample (ste = 0.01). The edge over other approaches may arise from incorporating the diversity of the locus within the human population as a prior in determining the alleles.

Among the approaches that report multiple solutions if ambiguities cannot be resolved, **HLA-VBSeq** performs best for WGS and WES, while **seq2HLA** performs best on RNAseq data, the respective datatypes they were designed for. These approaches are best suited to clinical applications where it is useful to rule out a specific detrimental genotype, rather than clinical scenarios, such as transplantation, where the patients’ exact HLA genotype needs to be determined.

On the requirement of data quality, we find that while the phase 3 WGS coverage of 2X is too low to produce reliable predictions (best four-digit accuracy was 66% by **HLA-VBSeq**), where there is sufficient coverage, performance between tools was inconsistent, highlighting that other factors inherent in the methods themselves impact performance.

We conclude that the maximum prediction accuracy of 86% for Class I + II may not be sufficient for clinical application; however, there is scope to improve the performance through an ensemble approach. Specifically, the higher performance from **OptiType** and **PHLAT** makes an approach attractive that harnesses the alternative assemblies in the new human reference build, hg38, and weights them according to the observed HLA allele frequency. This would enable construction of larger alleles that are anchored in the wider genomic context, as well as possible imputation of areas of low coverage owing to pseudogenes or other technical challenges in NGS. Furthermore, a recent study demonstrated that a large fraction of the alleles in the IMGT/HLA database are limited to a single population or individual [36]. Combining the genomic information from large-scale WGS studies will likely see a shift from allele to variant-based definitions of HLA genotypes.

In summary, as improvement in accuracy appears feasible, we expect that in the future, computational tools will be able to deliver fast economical HLA prediction from existing sequencing data.

**Key Points**

- On-demand *in silico* HLA genotyping offers an economical and efficient alternative to serology-based pathology tests.
- This article compares computational HLA typing methods on their accuracy and resource consumption based on >1000 samples with known HLA genotype.
- The highest accuracy for clinically relevant resolution (four digits) and scope (Class I + II) we observe is 81%.
- The fastest performance we observe was an average of 7 min per sample and the lowest memory consumption was on average 0.46 GB, however not by the same method.
- Clinical applications likely require higher accuracy, which may be achieved by an ensemble approach, such as using the alternative assemblies in the new human reference build and weighting them according to the observed HLA allele frequency.

## Supplementary data

Supplementary data are available online at http://bib.oxfordjournals.org/.

## Supplementary Material

Supplementary Data

## Notes

**Denis C. Bauer** is a team leader at CSIRO, with background in next-generation sequencing data analysis and machine learning. Her current research interests include medical genomics, SPARK-based variant association and genome engineering.

## Notes

**Armella Zadoorian** is an honours student at University of New South Wales, with background in medical science and genetics. Her current research interest includes molecular biology.

## Notes

**Laurence O. W. Wilson** is a postdoctoral fellow in the Transformational Bioinformatics team at CSIRO, with background in comparative genomics and epigenomics. His current research interests include computational genome engineering.

## Notes

**Natalie P. Thorne** is the Clinical Bioinformatics and Genomics Project Manager with the Melbourne Genomics Health Alliance, with background in statistics, bioinformatics and genetics. She currently improves the use of whole genome sequencing in the clinical setting.
