# Supplementary material for: Evaluation of computational programs to predict HLA genotypes from genomic sequencing data
Source: Brief Bioinform. 2016 Oct 31;19(2):179–87. doi: 10.1093/bib/bbw097 (PMC6019030; doi:10.1093/bib/bbw097)
Supplement: Supplementary Data [file bbw097_supplement.pdf]

0.1 Supplemental material

0.1.1 Software installation notes

The following software packages were investigated with installation notes below and observed error messages at the end of the document.

phlat Python-based and straight forward to set up and run.

seq2hla Python-based and straight forward to set up and run.

optitype Python-based and straight forward to set up and run.

hlavbseq Java-based code straight forward to set up and run. All genes processed as part of the package at the same time with probability assignment for allele confidence

hlaforest Perl-based code. Difficulties: Hardcoded paths that needed to be modified in two scripts

hlaminer Perl and bash based code. Difficulties: not designed for large sample numbers (outputs had hardcoded named files) other paths are also hardcoded (altered parseXMLblast.pl and HLAminer.pl)

hlareporter Largely Bash-based. Difficulties: requires fastq/bams in the same directory as code and has hardcoded folder structure. It predicts for one gene at a time. We could not get the example to work.

hlassign A collection of bash script instruction that are not bundled in a script. Difficulties: not designed for large sample numbers (requires manually copy of files that are not named systematically). We could not get the code to produce results

athlates Largely Bash-based. Difficulties: we needed to change bamtools index from BAMTOOLS to STANDARD in bam_manip.cpp for bamtools version 2.25.0. However, it still terminates with segmentation fault. Also only HLA-A .. C match up, the rest have no clean correspondence to the allele alignment files provided. While there are new alignment available (http://hla.alleles.org/data/) they do not work for HLA-C anymore.

omixon Available as GUI and the command line version has restrictions of how many samples can be run.

0.1.2 Extended tables

Tool	Digits	Class	Accuracy (Success)	Approximate Accuracy (Success)	Samples failed
optitype	2	I	81% (82%)		6
optitype	2	I+II	41% (82%)		6
optitype	4	I	71% (71%)		6
optitype	4	I+II	35% (71%)		6
hlavbseq	2	I	92% (92%)	97% (97%)	0
hlavbseq	2	I+II	72% (72%)	85% (85%)	0
hlavbseq	4	I	70% (70%)	82% (82%)	0
hlavbseq	4	I+II	52% (52%)	66% (66%)	0
hlaminer assembly	2	I	18% (53%)	21% (62%)	19
hlaminer assembly	2	I+II	27% (59%)	29% (62%)	19
hlaminer assembly	4	I	10% (29%)	18% (52%)	19
hlaminer assembly	4	I+II	17% (36%)	23% (49%)	19
hlaminer alignment	2	I	11% (63%)	11% (63%)	0
hlaminer alignment	2	I+II	27% (47%)	27% (47%)	0
hlaminer alignment	4	I	5% (30%)	7% (40%)	0
hlaminer alignment	4	I+II	15% (26%)	20% (35%)	0
phlat	2	I	56% (70%)		0
phlat	2	I+II	53% (64%)		0
phlat	4	I	40% (50%)		0
phlat	4	I+II	38% (46%)		0
seq2hla	2	I	14% (14%)	14% (41%)	0
seq2hla	2	I+II	13% (21%)	13% (44%)	0
seq2hla	4	I	8% (8%)	10% (30%)	0
seq2hla	4	I+II	7% (12%)	9% (32%)	0

Table 1: **Extended accuracy table WGS data.**

Tool	Digits	Class	Accuracy (Success)	Approximate Accuracy (Success)	Samples failed
optitype	2	I	99% (100%)		1
optitype	2	I+II	50% (100%)		1
optitype	4	I	98% (98%)		1
optitype	4	I+II	49% (98%)		1
hlavbseq	2	I	97% (97%)	100% (100%)	0
hlavbseq	2	I+II	85% (85%)	89% (89%)	0
hlavbseq	4	I	86% (86%)	95% (95%)	0
hlavbseq	4	I+II	68% (68%)	77% (77%)	0
hlaminer assembly	2	I	73% (79%)	76% (83%)	0
hlaminer assembly	2	I+II	62% (71%)	64% (73%)	0
hlaminer assembly	4	I	50% (54%)	66% (72%)	0
hlaminer assembly	4	I+II	43% (49%)	53% (61%)	0
hlaminer alignment	2	I	64% (65%)	64% (65%)	0
hlaminer alignment	2	I+II	56% (57%)	56% (58%)	0
hlaminer alignment	4	I	26% (26%)	46% (46%)	0
hlaminer alignment	4	I+II	26% (27%)	42% (43%)	0
phlat	2	I	93% (93%)		0
phlat	2	I+II	81% (81%)		0
phlat	4	I	88% (88%)		0
phlat	4	I+II	73% (73%)		0
seq2hla	2	I	89% (89%)	89% (89%)	0
seq2hla	2	I+II	78% (78%)	78% (78%)	0
seq2hla	4	I	71% (71%)	81% (81%)	0
seq2hla	4	I+II	60% (61%)	71% (71%)	0

Table 2: **Extended accuracy table WES data.**

Tool	Digits	Class	Accuracy (Success)	Approximate Accuracy (Success)	Samples failed
optitype	2	I	100% (100%)		0
optitype	2	I+II	50% (100%)		0
optitype	4	I	99% (99%)		0
optitype	4	I+II	50% (99%)		0
hlavbseq	2	I	86% (86%)	100% (100%)	0
hlavbseq	2	I+II	81% (81%)	90% (90%)	0
hlavbseq	4	I	80% (80%)	98% (98%)	0
hlavbseq	4	I+II	67% (67%)	80% (80%)	0
hlaminer assembly	2	I	79% (86%)	80% (86%)	0
hlaminer assembly	2	I+II	68% (79%)	68% (79%)	0
hlaminer assembly	4	I	57% (62%)	72% (78%)	0
hlaminer assembly	4	I+II	52% (61%)	61% (71%)	0
hlaminer alignment	2	I	59% (59%)	59% (59%)	0
hlaminer alignment	2	I+II	52% (52%)	52% (52%)	0
hlaminer alignment	4	I	20% (20%)	33% (33%)	0
hlaminer alignment	4	I+II	20% (20%)	30% (30%)	0
phlat	2	I	98% (98%)		0
phlat	2	I+II	86% (86%)		0
phlat	4	I	96% (96%)		0
phlat	4	I+II	81% (81%)		0
seq2hla	2	I	98% (98%)	98% (98%)	0
seq2hla	2	I+II	85% (85%)	85% (85%)	0
seq2hla	4	I	95% (95%)	97% (97%)	0
seq2hla	4	I+II	79% (79%)	81% (81%)	0

Table 3: **Extended accuracy table RNAseq data.**

0.1.3 Coverage per dataset

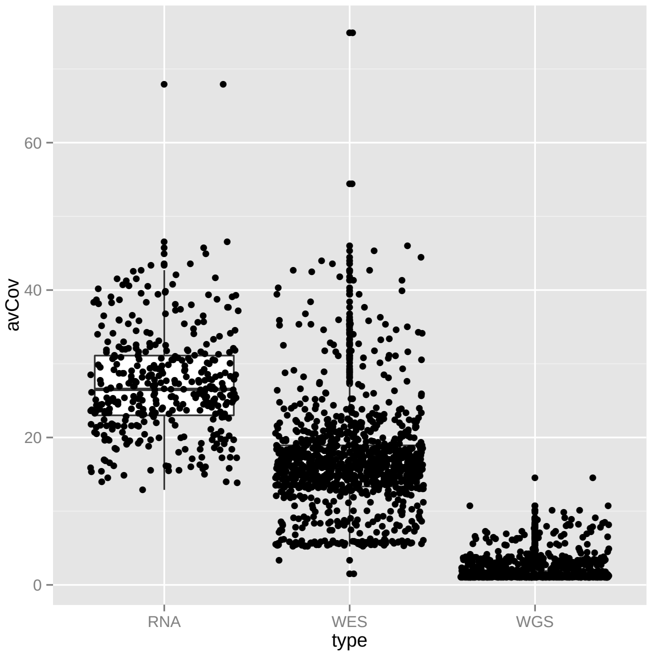

Figure 1: Coverage information per dataset.

0.1.4 Certain samples

Dataset	mean (ste)	max	min
Full	0.26 (0.01)	1	-0.17
Certain	0.22 (0.01)	1	-0.44

Table 4: Comparison of correlations between full and samples with certain genotype. There are 37 samples with certain genotype.

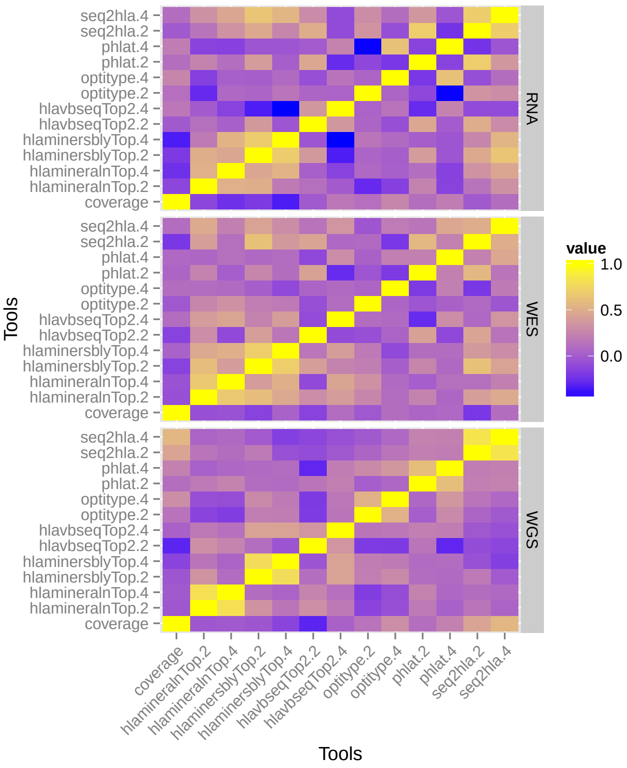

Figure 2: Coverage vs accuracy on the samples with certain genotype.

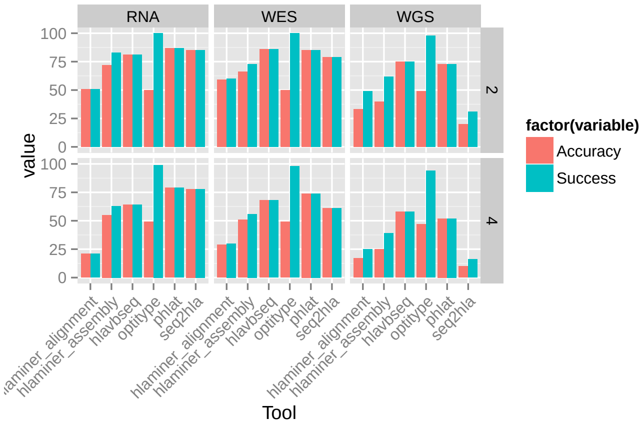

Figure 3: Accuracy and success on the samples with certain genotype.

0.1.5 Error messages

Tool	error msg	count WGS	count WES	count RNAseq
optitype	unpaired weight	4	1	0
	optimisation	2	0	0
hlaminer assembly	Illegal division by zero	12	0	0
phlat	mapping error	7	0	0

0.1.6 OptiType unpaired weight Example

```
1:25:56.36 42 reads loaded. Creating dataframe...
1:25:56.36 Dataframes created. Shape: 42 x 11179, hits: 11098 (28139), sparsity: 1 in 16.69
1:25:56.38 Alignment pairing completed. 10 paired, 126 unpaired, 0 discordant
Traceback (most recent call last):
  File "/flush2/bau04c/apps/optitype/1.0/OptiTypePipeline.py", line 305, in <module>
    "in your config file (currently %.3f), because you may need to resort to using unpaired
         reads.") % unpaired_weight
TypeError: not enough arguments for format string
```

0.1.7 OptiType optimisation Example

```
Deterministic time = 0.09 ticks (0.11 ticks/sec)

CPLEX> Incumbent solution written to file '/flush1/tmpdir/ruby_inter/bau04c.629683/tmpfo8saB.
     cplex.sol'.
CPLEX> Traceback (most recent call last):
  File "/flush2/bau04c/apps/optitype/1.0/OptiTypePipeline.py", line 374, in <module>
    result = op.solve(args.enumerate)
  File "/flush2/bau04c/apps/optitype/1.0/model.py", line 184, in solve
    self.__instance.c.add(expr >= 1)
  File "/apps/python/2.7.10/lib/python2.7/site-packages/pyomo/core/base/constraint.py", line
      1195, in add
    cdata = self._check_skip_add(self._nconstraints + 1, expr)
  File "/apps/python/2.7.10/lib/python2.7/site-packages/pyomo/core/base/constraint.py", line
      895, in _check_skip_add
    self._data[index].cname(True)))
ValueError: Invalid constraint expression. The constraint expression resolved to a trivial
     Boolean (False) instead of a Pyomo object. Please modify your rule to return Constraint.
     Infeasible instead of False.

Error thrown for Constraint 'c[1]'
```

0.1.8 PHLAT mapping error

```
3922839 reads; of these:
  3922839 (100.00%) were paired; of these:
    3885014 (99.04%) aligned concordantly 0 times
    16051 (0.41%) aligned concordantly exactly 1 time
    21774 (0.56%) aligned concordantly >1 times
0.96% overall alignment rate
.....Process Bowtie 2 mapping on HG00278_lc.......

.....Prepare files of HG00278_lc for PHLAT.......

..... Running PHLAT .......
!!!ERROR encountered: please make sure bowtie mapping outpur is good,e.g. obtain good mapping
     rate

.....Done! Total PHLAT process time:0:00:00.134314.....
```
